# Electrode array design determines scalar position, dislocation rate and angle and postoperative speech perception

**DOI:** 10.1007/s00405-021-07160-2

**Published:** 2021-11-15

**Authors:** Manuel Christoph Ketterer, Antje Aschendorff, Susan Arndt, Rainer Beck

**Affiliations:** Department of Otorhinolaryngology-Head and Neck Surgery, Faculty of Medicine, Medical Center-University of Freiburg, University of Freiburg, Killianstrasse 5, 79106 Freiburg, Germany

**Keywords:** Cochlear morphology, Electrode array design, Scalar position, Coverage, Speech perception

## Abstract

**Purpose:**

The aim of this study is to examine the scalar dislocation rate in straight and perimodiolar electrode arrays in relation to cochlear morphology. Furthermore, we aim to analyze the specific dislocation point of electrode arrays depending on their design and shape and to correlate these results to postoperative speech perception.

**Methods:**

We conducted a comparative analysis of patients (ears: *n* = 495) implanted between 2013 and 2018 with inserted perimodiolar or straight electrode arrays from Cochlear™ or MED-EL. CBCT (cone beam computed tomography) was used to determine electrode array position (scalar insertion, intra-cochlear dislocation, point of dislocation and angular insertion depth). Furthermore, cochlear morphology was measured. The postoperative speech discrimination was compared regarding electrode array dislocation, primary scalar insertion and angular insertion depth.

**Results:**

The electrode array with the highest rate of primary SV insertions was the CA; the electrode array with the highest rate of dislocations out of ST was the Flex^Soft^. We did not find significantly higher dislocation rates in cochleostomy-inserted arrays. The angle of dislocation was electrode array design-specific. A multivariate nonparametric analysis revealed that the dislocation of the electrode array has no significant influence on postoperative speech perception. Nevertheless, increasing angular insertion depth significantly reduced postoperative speech perception for monosyllables.

**Conclusion:**

This study demonstrates the significant influence of electrode array design on scalar location, dislocation and the angle of dislocation itself. Straight and perimodiolar electrode arrays differ from each other regarding both the rate and place of dislocation. Insertion via cochleostomy does not lead to increased dislocation rates in any of the included electrode arrays. Furthermore, speech perception is significantly negatively influenced by angular insertion depth.

## Introduction

Cochlear implant (CI) surgery focusses more and more on the impact of cochlear morphology and consequently on intra-cochlear electrode array position and postoperative speech perception. Previous studies described different ways of estimating cochlear morphology preoperatively. Escudé et al. [1] established distance A as the distance from the round window (= RW) to the lateral wall and a perpendicular distance B, both intersecting the modiolus. Ketterer et al. [2] described a third measure, the cochlear height and the fact that the electrode array was more likely to dislocate within a cochlea of smaller height and smaller diameter. Aschendorff et al. [3] first examined scalar position via rotational tomography for patients inserted with a Contour (*n* = 21) versus a Contour Advance (*n* = 22) (= CA) electrode array and described significantly higher speech discrimination results for scala tympani (ST) compared to scala vestibuli (SV) insertion. In a linear regression analysis for 14 of the 15 patients described by Skinner et al. [4], Finley et al. [5] calculated that scalar position, age at implantation and total number of electrode contacts within the SV accounted for 83% of the variance in monosyllabic word scores. Holden et al. [6] described that the position of electrode arrays closer to the modiolus was positively correlated with the outcome. As a result, scalar position detection of the electrode array via CBCT (cone beam computed tomography) or HRCT (high resolution computed tomography) should be a consideration in postoperative quality control to provide important feedback to the surgeon [3]. The goal of this study is to examine the scalar dislocation rate in both straight and perimodiolar electrode arrays. To the best of our knowledge, this is the first large cohort study analyzing the specific position of dislocation of electrode arrays depending on their design and shape. Furthermore, we aimed to evaluate the impact of scalar dislocation, electrode array design and angular insertion depth on postoperative speech perception.

## Methods

### Study and subject

We performed a retrospective analysis of adult patients implanted between 2013 and 2018. HRCT and magnetic resonance imaging had been conducted preoperatively and patients with cochlear anomalies and signs of sclerosis or obliteration were excluded from this study. We only included patients inserted with a Cochlear™ Contour Advance^®^ (CI24RECA, CI412/512/612) (= CA), Cochlear™ slim straight^®^ (422/522/622) (= SSA) or Cochlear™ slim modiolar^®^ (532/632) electrode array (= SMA) (Cochlear Limited, NSW, Sydney, Australia) and MED-EL Flex^24^, MED-EL Flex^28^ and MED-EL Flex^Soft^ (MED-EL, Innsbruck, Austria). Electrode arrays were inserted via cochleostomy (= CS), round window (= RW) and extended round window (= ERW) insertion. The patient chose the manufacturer following individual consulting. If the patient chose MED EL and showed residual hearing, the FLEX^24^ was used in most cases. In patients implanted with a device from Cochlear™, the SSA or the SMA was used in patients with residual hearing, otherwise the CA was also used quite often due to the later availability of the SSA and the SMA.

### Radiological evaluation

Postoperative imaging was performed using a DynaCT-equipped Axium Artis dTA angiography unit (Siemens Co., Erlangen, Germany) with a digital flat-panel detector [3, 7]. Two experienced head and neck surgeons and two head and neck radiologists independently analyzed the scans regarding scalar electrode position (ST versus SV insertion, intracochlear dislocation, angular insertion depth) and cochlear size (diameters in length and width) and used Impax 6 from Agfa Healthcare for reconstruction. The scans were not evaluated by the surgeons who, performed the CI surgery but by independent and experienced head and neck surgeons to reduce bias. All included electrode arrays were fully inserted. Cochlear size was evaluated in distance A from the round window to the lateral wall through the modiolus and perpendicular distance B [1, 2, 8]. The angular insertion depth was evaluated between the vectors of distance A and the distance through the bloom artefact of the apical electrode and the modiolus as described before [2, 8, 9]. Dislocation analysis and analysis of scalar position were performed on three-dimensionally reconstructed cross-sectional images as previously described [2, 8], i.e. the 3D-reconstruction could be rotated and browsed in whichever direction the specialists needed to come to their respective conclusion. Every image with discrepancy was reviewed and discussed interdisciplinary until a final agreement and measurement was achieved.

We compared preoperative HRCT scans to postoperative CBCT scans to examine the hypothesis that straight electrode arrays could lead to a mismatch of cochlear morphology measurements due to their more lateral electrode artifacts.

### Audiological evaluation

Open set speech perception is evaluated regularly in a soundproof chamber in a standard clinical setting using the Freiburg numbers and the Freiburg monosyllables test both with presentation at a volume of 65 dB SPL in quiet. Speech discrimination is scored as percentage correct. The audiologists conducting speech perception were blinded and did not know scalar position or dislocation analysis.

### Statistics

Statistical analysis was performed using Gnu R statistical computation and graphics system (GNU R, Version 3.6.2, Core Team, Vienna, Austria, http://www.R-project.org), extended with the packages NLME (Linear and Nonlinear Mixed Effects Models, Version 3.1, Pinheiro et al., https://CRAN.R-project.org/package=nlme) and ggplot2 (Version 3.3.1, Hadley Wickham, https://ggplot2.tidyverse.org). Where applicable, ANOVA and Tukey’s Honest were used. Nonlinear mixed effect models were applied for the analysis of speech discrimination and compared directly by ANOVA and AIC. For array comparisons, the residuals were analyzed using pairwise Mann–Whitney *U* tests with adjustment by Holm. Results were calculated descriptively and are shown in the text and in tables as mean, standard deviation, maximum and minimum. The level of significance was set at 5.0%.

### Ethics committee

This retrospective study took place in the Department of Otorhinolaryngology, Head and Neck Surgery at the Implant Center of the University Hospital Freiburg. The study was approved by the hospital´s Ethics Committee according to the Declaration of Helsinki (Washington, 2002) (Number of Ethics Committee approval: 406/19) and registered in the German Clinical Trials Register (http://www.drks.de/DRKS00019807).

## Results

### Study, subject and cochlear morphology

**Fig. 1:**
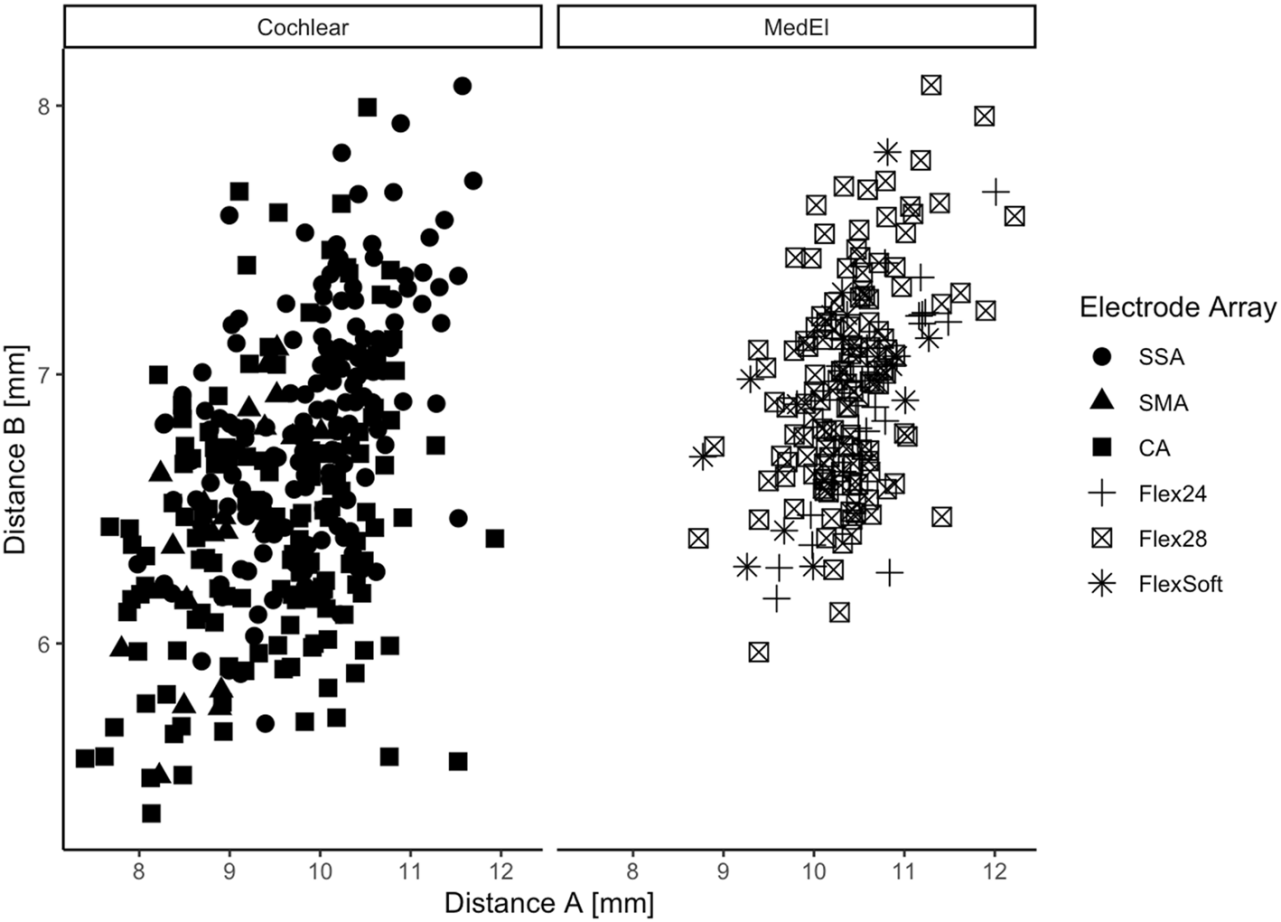
Left: Regarding electrode arrays from Cochlear™ (CA = Contour Advance; SMA = CI 532/632 = slim modiolar array; SSA = CI 422/522/622 = slim straight array) the use of the CA is significantly more frequent in cochleae with a smaller size of the cochlear basal turn (product of distance A and B). Right: We could not find significance for the cochlear basal turn product between the different electrode arrays of MED-EL

We included 495 ears implanted between 2013 and 2018. We included 40 bilaterally implanted and 415 unilaterally implanted patients. 259 left and 236 right cochleae; 327 ears, implanted with a device from Cochlear™ and 168 ears implanted with a device from MED-EL were included. The mean age was 52.7 years and the most-often inserted electrode array was the SSA with 32.7% (see Table 1: distribution of analyzed electrode arrays). The measurements of the diameters of the cochlear basal turn confirm previous studies [1, 2], calculating mean distance A with 9.92 mm and distance B with 6.74 mm (see Table 2). Regarding the electrode array portfolio of Cochlear™, the cochlear basal turn size (product of distance A and B) shows significant impact on the surgeon’s electrode array choice (see Fig. 1). Differences between the two manufacturers regarding the cochlear basal turn product were not analyzed due to patient’s preoperative choice of the manufacturer. The use of the CA is significantly more frequent in cochleae with a smaller cochlear basal turn product of distance A and B compared to the SMA and the straight electrode (SSA) array of Cochlear™ (CA versus SSA: *p* < 0.0001; SMA versus SSA: *p* = 0.0025). Nevertheless, we did not find significance for the cochlear basal turn product between the different electrode arrays of MED-EL.

**Table 1:** Synopsis of study group (in total: *n* = 495)

Manufacturer (*n*)	Cochlear™: 327 MED-EL: 168
Electrode array (*n*)	Contour Advance (Cochlear™) (= CA): 143 CI 422/522/622 (Cochlear™) (= SSA): 162 CI 532/632 (Cochlear™) (= SMA): 22 Flex^24^ (MED-EL): 129 Flex^28^ (MED-EL): 24 Flex^Soft^ (MED-EL): 15
Side (*n*)	Left: 259 Right: 236
Age	52.7 years (min 18.0; max 86.2)

**Table 2:** Cochlear measurements (distance A and B), insertion angle, scalar position in total (SD = standard deviation) and distribution of the insertion technique

	Mean	SD	Minimum	Maximum
Distance A (mm)	9.92	0.86	7.4	12.2
Distance B (mm)	6.74	0.49	5.4	8.1
Insertion angle (°)	418.8	103.7	199	794
Scalar position in total (*n*)	ST: 434 (87.7%) TD: 32 (6.5%) SV: 25 (5%) VD: 4 (0.8%)			
Insertion technique in total (*n*) and percentage (%)	Electrode array	CS	RW	ERW
	CA (Cochlear™)	140/98%	3/2%	/
	SSA (Cochlear™)	47/29%	110/68%	5/3%
	SMA (Cochlear™)	12/54.5%	10/45.5%	/
	Flex^24^ (MED-EL)	21/16.3%	102/79.2%	6/4.2%
	Flex^28^ (MED-EL)	10/41.6%	13/54.2%	1/4.2%
	Flex^Soft^ (MED-EL)	12/80%	3/20%	/

*CS* cochleostomy, *RW* round window, *ERW* extended round window

We excluded the hypothesis that the SSA could lead to larger cochlear measurements due to the lateral electrode array artifacts. Therefore, we compared preoperative HRCT scans to postoperative CBCT scans. We evaluated both scans blinded and independently and did not find different measurements for either distance A or B.

Furthermore, we compared cochlear size to dislocation behavior and could not detect significant differences for cochlear distance A or B compared dislocated or non-dislocated electrode arrays.

### Angular insertion depth and dislocation manner

**Fig. 2:**
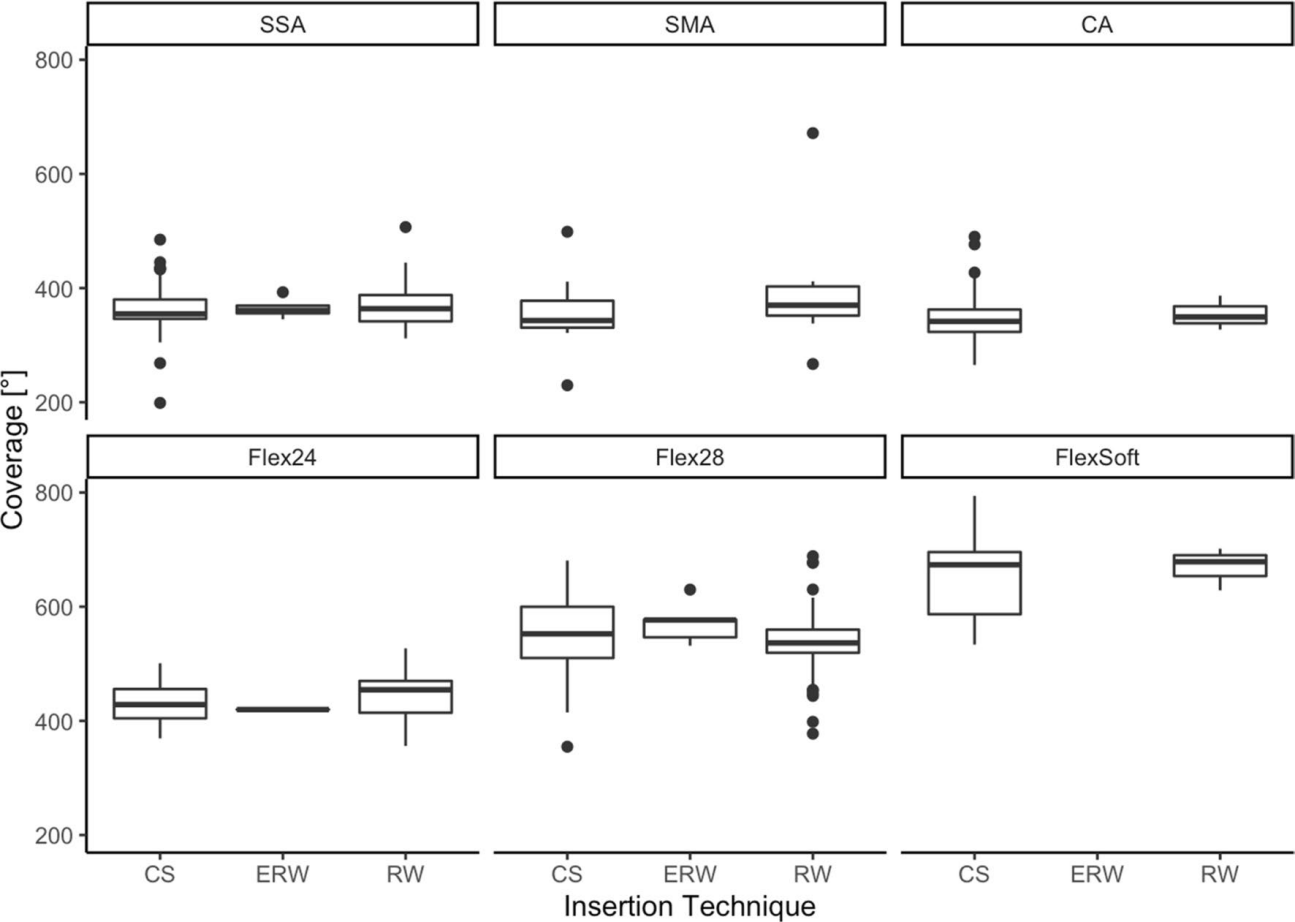
Angular insertion depth depends on electrode array design and correlates to electrode array length. Insertion technique has no significant influence on electrode array-specific angular insertion depth (CS = cochleostomy; RW = round window; ERW = extended round window)

Figure 2 shows the mean angular insertion depth for each included electrode array. Regarding the included Cochlear™ electrode arrays, we measured a significantly higher angular insertion depth for the SSA compared to the CA (*p* = 0.0004). The angular insertion depth was comparable between the CA and the SMA (*p* = 0.15) or between the SSA and the SMA (*p* = 0.9996). Regarding the electrode arrays from MED-EL, the Flex^24^ showed significantly lower angular insertion depth compared to the longer electrode arrays Flex^28^ and Flex^Soft^ (*p* < 0.00001) as expected. All included electrode arrays from Cochlear™ showed significantly shorter angular insertion depth than the electrode arrays from MED-EL (*p* in all comparisons < 0.0065) (see Fig. 2).

**Fig. 3:**
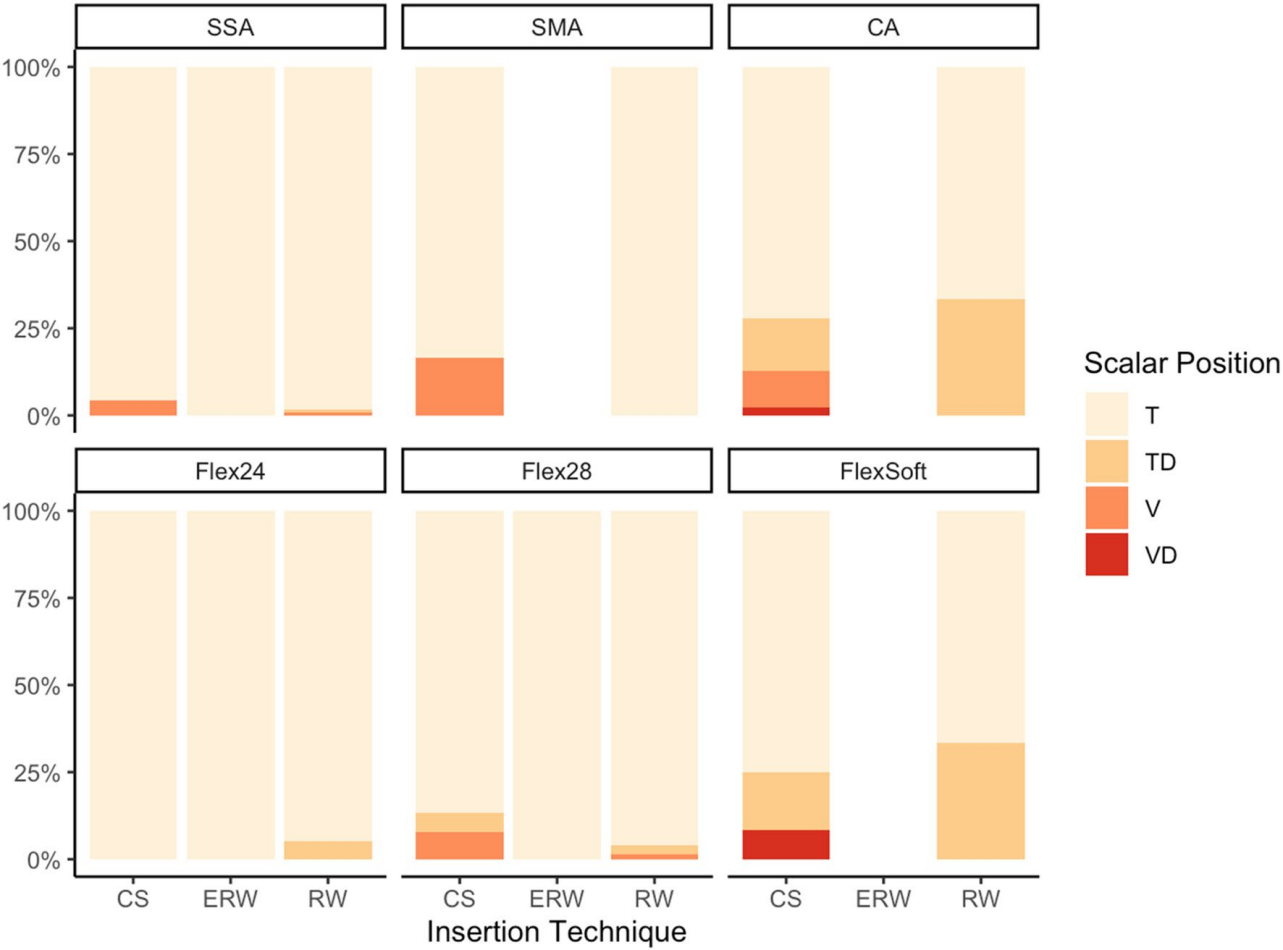
Count of primary scala tympani insertions (T), dislocations out of ST (TD), scala vestibuli insertions (V) and dislocations out of scala vestibuli (VD) for each examined electrode array

In regard to insertion types, the electrode arrays behaved distinctively in this cohort: CA exhibited the highest rate of SV insertions [SV: 16.1%; TD (dislocation out of ST): 15.4%]; the electrode array with the highest rate of ST dislocations was the Flex^Soft^ (SV 6.7%; TD 20.0%). The SMA showed no dislocations; the SSA only one dislocation out of ST and 3 SV insertions via CS (SV 1.9%; TD 0.6%) (see Fig. 3). An SV insertion was defined as a direct insertion into SV. SV insertions mostly occurred in CS approaches and most frequently for the CA electrode array (see Fig. 3). As SV insertions mostly depend on the position of the CS, they are only partially influenced by array design.

Table 2 and Fig. 3 show that electrode arrays included in this study were inserted via CS and RW and in some cases via ERW. Comparing angular insertion depth in all approaches (ANOVA Tukey post-hoc), we could not find any significant impact.

**Fig. 4:**
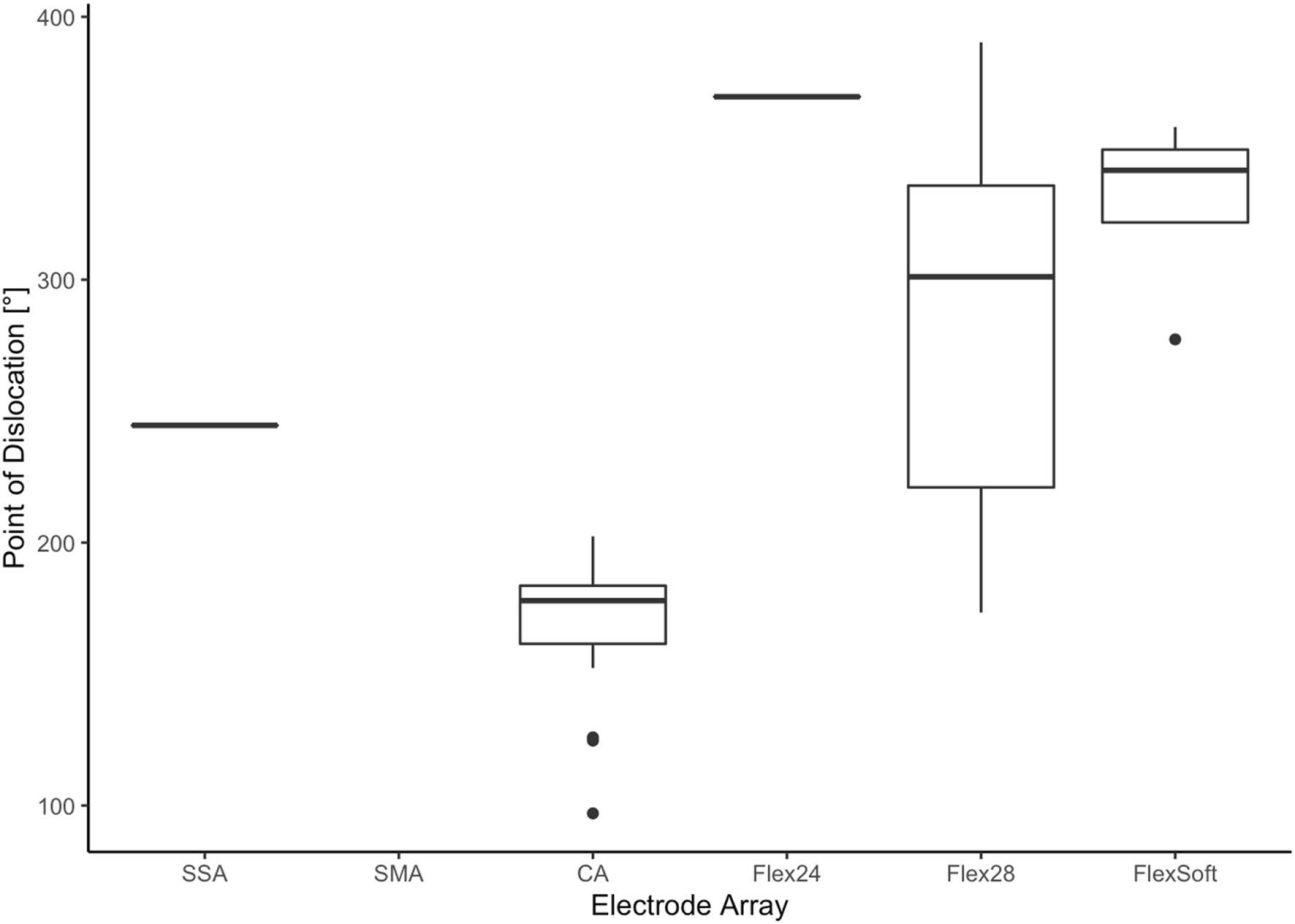
The angle of dislocation is electrode-array-specific

Measuring the specific position of dislocation for each electrode array, we found that the position of dislocation depends on the electrode array itself (see Fig. 4). We measured a significant lower point of dislocation for the CA compared to both the Flex^28^ (*p* < 0.00001) and the Flex^Soft^ (*p* < 0.00001). The point of dislocation is electrode-design specific. Perimodiolar electrode arrays dislocate between 160 and 180° (CA: mean ± SD: 170 ± 25°), whereas straight electrode arrays dislocate between 280° and 330° (Flex^28^: mean ± SD: 284 ± 87°; Flex^Soft^: mean ± SD: 330 ± 36°).

### Impact on speech perception

**Fig. 5:**
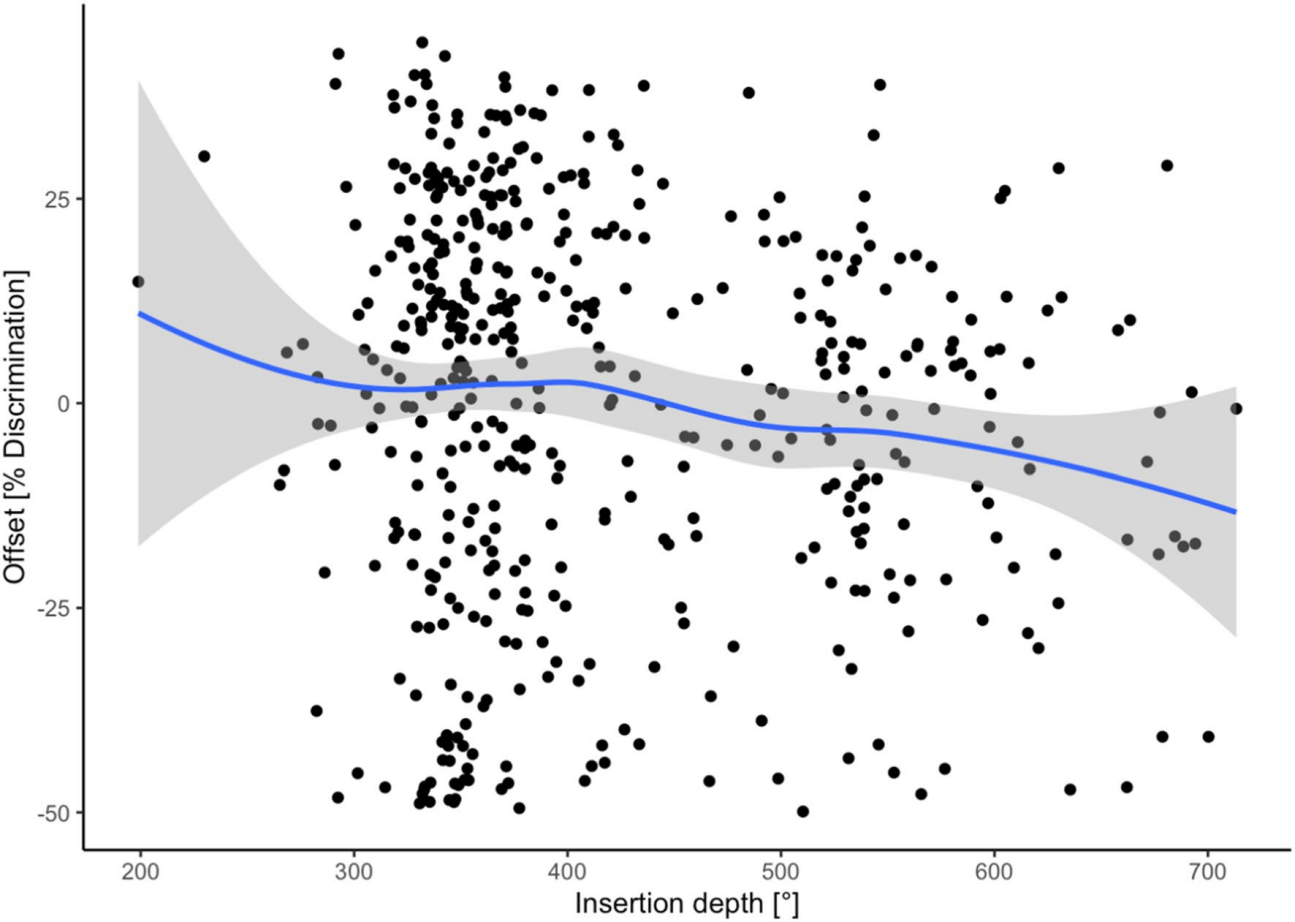
Plotting individual speech discrimination versus the insertion depth shows a significant negative effect of deeper angular insertion with regard to speech discrimination. The *y* axis depicts the influence of angular insertion depth as offset in speech discrimination compared to the model that does not comprise angular insertion depth as a factor

Data could not be acquired for 11 ears for the evaluation of speech discrimination, therefore 484 ears were included. The Freiburg Number discrimination test showed a considerable ceiling effect over all measurements in all patients (1st quartile 80%, mean 82.25%, 3rd quartile 100%) and was therefore discarded in regard to further analysis. The Freiburg Monosyllable test showed a more homogeneous distribution (1st quartile 20%, mean 43.9%, 3rd quartile 70%). After constructing an asymptotic growth model for every individual ear, the fitness of different models could be compared using ANOVA. Including angular insertion depth could improve the fitness of the model significantly when including all electrode arrays (*p* < 0.0001), but not when comparing electrode arrays from Cochlear™ and MED-EL separately. Pooling all electrode arrays, there was a significant (*p* < 0.0001) but very small effect favoring shorter electrode arrays (see Fig. 5). In regard to primary insertion into SV or ST and in regard to dislocated or non-dislocated electrode arrays, the models showed no higher or lower speech perception results (pooled and manufacturers separately).

**Fig. 6:**
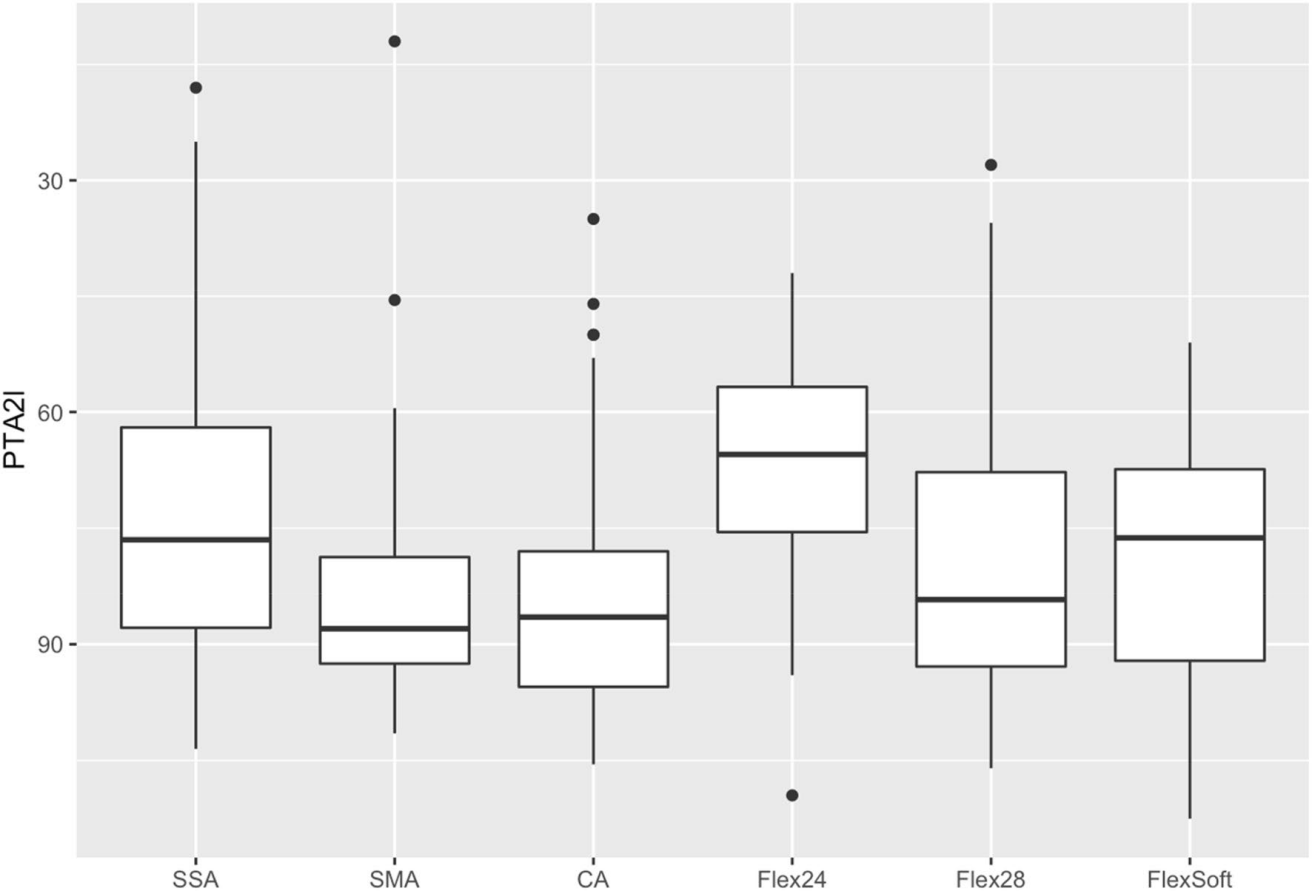
Preoperative residual hearing levels expressed as PTA 2 (250 and 500 Hz) for each included electrode array

Comparing the residual hearing of the different electrode arrays, expressed as PTA 2 (250 and 500 Hz), the tendency towards shorter electrode arrays in patients with better residual hearing can be seen (see Fig. 6). How the surgeon chooses the respective length of the electrode array should be examined further.

## Discussion

### Study, subject and cochlear morphology

This is the largest study (*n* = 495 ears) so far evaluating the influence of cochlear morphology and electrode array design on electrode array position. We measured the cochlear basal turn with a mean distance A of 9.92 mm and distance B of 6.74 mm (see Table 2). Once more, we can now confirm the data published so far [1, 2, 10]. Furthermore, this is the first study describing that the CA was more frequently implanted in smaller cochlea with less basal turn size (product of distance A and B) (see Fig. 1). The surgeon usually chooses the CA electrode array in patients without residual hearing to be as close to the modiolus as possible. Furthermore, the CA is usually not the first electrode array choice to preserve residual hearing due to its rigidity and larger diameter.

### Angular insertion depth and dislocation manner

We could measure specific angular insertion depth for each included electrode array (see Fig. 2). Previous studies [1, 2] showed that the angular insertion depth is dependent on the cochlear size. This work extends the earlier studies by defining electrode array-specific angular insertion depth, depending on both electrode array design and cochlear morphology.

This study demonstrates specific dislocation behavior of each examined electrode array (see Fig. 3). The electrode array with the highest rate of ST dislocations was the Flex^Soft^ array. The electrode array with the highest rate of SV insertions was the CA due to insertion via CS. Ketterer et al. [2] showed that dislocation depends on cochlear morphology and that a smaller cochlear height is a risk factor for SV insertion and dislocation for CS-inserted CA electrode arrays. Nevertheless, Aschendorff et al. [11] described individual learning curves of the surgeon and dislocation rates also depend on the surgeon’s experience not only on electrode array design and cochlear morphology.

The SMA did not dislocate in any of the included patients in this study. Aschendorff et al. [12] described in a multi-center study that all patients (*n* = 44) implanted with the SMA from Cochlear™ exhibited a complete ST insertion without dislocation in round window and cochleostomy approaches. We can now confirm that the SMA is the electrode array without any dislocation and seems to be very well designed for staying within the initial inserted cochlear scala.

We could show that the position of dislocation is electrode-design specific (see Fig. 4) and depends on electrode array design itself. The SSA has a stiff internal stylet and is a lateral wall array. Therefore, the dislocation point is more apical than the dislocation point of the perimodiolar CA. The CA is inserted via an Advance Off–Stylet™ insertion technique and due to its preformed perimodiolar design the point when dislocation might happen is earlier and at approximately 180°. Further studies described the ascending cochlear basal turn at around 180° as sensitive for scalar dislocation [13–15]. Aschendorff et al. [7] speculated that perimodiolar electrode arrays may touch the outer cochlear wall at 180° while rotating with an upward direction and being pushed forward, resulting in perforation of the basilar membrane.

With a maximal active length of 25 mm (source: Cochlear™), the SSA is shorter than the straight Flex^28^ and Flex^Soft^ and shows less insertion depth. MED EL electrode arrays do not have such a rigid internal stylet as the SSA. Therefore, the point of dislocation is higher in longer and more flexible MED EL Flex^28^ and Flex^Soft^ due to their flexibility and trajectory. Furthermore, the height of the ST decreases within the ascending part of the basal turn towards the apical cochlear part [16]. Therefore, long electrode arrays like the Flex^28^ and Flex^Soft^ showed increasing risk of dislocation in the apical cochlear part. The fact that the Flex^24^ showed the highest dislocation point is interesting but since there was only one dislocated Flex^24^ array further studies are required.

Boyer et al. [13] analyzed 61 CBCT scans of 54 patients. Eight perimodiolar electrode arrays and one straight electrode array were described as dislocated. The authors speculated that straight electrode arrays dislocate at approximately 370°, whereas perimodiolar electrode arrays dislocate at around 170°–190°. We can now confirm that the CA dislocates at approximately 170°, corresponding to the ascending part of the cochlear basal turn. Boyer et al. [13] compared only two groups: perimodiolar (CI 512 and CI24RECA) versus straight (Flex^Soft^, Flex^24^, Flex^28^ and Flex^Standard^) electrode arrays. Whereas their defined perimodiolar group seems to be a homogeneous electrode array cohort (*n* = 31), the straight electrode array group (*n* = 30) is not only too small to define angular insertion depth and manner of dislocation but also inhomogeneous, comparing electrode arrays from MED-EL of different lengths and diameters. Furthermore, they neither excluded the CS-inserted electrode arrays nor calculated if there is a statistically relevant effect of RW versus CS or not. We examined the influence of the insertion location and can show that CS does not lead to higher dislocation rates or SV insertions in any of the included arrays. Boyer et al. and Wanna et al. [13, 17] described that straight electrode arrays are more often completely inserted within the ST and hypothesized that straight electrode arrays are more flexible due to the silicon density of the electrode array. Nevertheless, they did not exclude CS-inserted electrode arrays. Additionally, the CA electrode array, which was originally designed for CS approach, was inserted via RW. Rebscher et al. and Souter et al. [18, 19] described that the CA is only designed for CS due to the higher incidence of cochlear trauma in RW approach. They argued that the electrode array may be too close to the lateral wall, which might result in traumatic deflection. Therefore, the insertion of the CA via CS is recommended, even though RW insertions are also possible in exceptions. Table 2 shows that with only a few exceptions, the included CA arrays of our study were not inserted via RW, but in 98% via CS. In conclusion, this study extends the previous knowledge of angular insertion depth, dislocation behavior and the influence of cochlear morphology. Furthermore, we could measure defined angular insertion depth and dislocation data of each included electrode array and could show that each electrode array has a specific position of dislocation.

### Impact on speech perception

Electrode array design and its influence on speech perception is still a disputed topic. This study demonstrates that speech perception may be negatively influenced by the electrode array’s angular insertion depth. Scalar dislocation has no significant impact on postoperative speech perception. Perimodiolar electrode arrays have been described as closer to the spiral ganglion cells with reduced spread of excitation [12, 20] and situated closer to the target spiral ganglion cells, therefore requiring lower stimulation levels than the lateral wall straight electrode arrays. That might provide better speech perception results [12]. Holden et al. [6] reported that the position of electrode arrays closer to the modiolus was positively correlated with the outcome. Nevertheless, other studies reported lower speech discrimination levels for perimodiolar electrode arrays compared to straight electrode arrays [21, 22]. We did not observe different speech discrimination between the groups of perimodiolar and straight electrode arrays. But we could show that the number of dislocations and of SV insertions depends on the electrode array itself. Furthermore, this study demonstrates that the angular insertion depth negatively impacts on speech perception results. Previous studies showed different results examining the influence of angular insertion depth on postoperative outcome [23–26]. Finley et al. [5] examined 14 patients, implanted with a device from Advanced Bionics™, and reported that lower outcome scores are associated with greater angular insertion depth and greater number of contacts located in SV. They speculated that the scalar dislocation compromises neural pathways by damaging the basilar membrane and spiral ganglion. Holden et al. [6] (*n* = 114) described that the CNC final score was higher in patients with more electrodes located in ST compared to SV. Finley et al. and Holden et al. [5, 6] speculated that SV inserted electrode arrays can lead to pitch confusion and diminished speech recognition due to cross-turn stimulation, but included different types of electrode arrays and did not calculate their results electrode array-specific. Baskent and Shannon [27] examined MED-EL recipients and manipulated electrical stimulation via deactivation of apical electrodes. They described no further benefit for active electrodes over an angular insertion depth of 360°. James et al. [28] collected radiological data via computed tomography and described a negative correlation of angular insertion depth and speech recognition with statistical significance (*p* < 0.001) but detected a very weak correlation (*r*^2^ = 0.09). However, they included only 96 patients with 9 different electrode arrays. We can now confirm their results regarding the negative impact of increasing angular insertion depth on speech perception in one of the largest cohort studies examining the influence of electrode array design on scalar location, dislocation and the position of dislocation. James et al. [28] described that less insertion depth is associated with better residual hearing preservation [29, 30]. We speculate that the negative correlation of speech perception with increasing angular insertion depth is due to cross-talk of the electric fields in the apical scalae. Nevertheless, there are also other factors to mention, such as the cochlear morphology. As the suspicious lack of publications shows, there is still a lot for speculation and discussion.

The included number of SMA and Flex^Soft^ arrays in this study was lower compared to the other included electrode arrays and therefore statistical analysis was more difficult. The dislocation rates and SV insertion rates via CS of the included CA group are lower in this study compared to previous studies [28]. Therefore, there might be a sampling bias regarding the influence analysis of scalar dislocation and SV insertion on speech perception results. Further research should be multi-centric to examine more of these electrode arrays. Nevertheless, in conclusion, this is the only study with statistical power and analysis of each electrode array separately, without the bias of electrode arrays differing with respect to length, diameter and rigidity.

## Abbreviations

ST: Insertion in scala tympani
SV: Insertion in scala vestibuli
TD: Tympani dislocation = dislocation out of ST
VD: Vestibuli dislocation = dislocation out of SV
CBCT: Cone beam computed tomography
HRCT: High-resolution computed tomography
CI: Cochlear implant/cochlear implantation
CA: Cochlear™ Contour Advance^®^ electrode array
SMA: Cochlear™ slim modiolar^®^ electrode array
SSA: Cochlear™ slim straight^®^ electrode array
Flex^24^: MED-EL Flex^24^ electrode array
Flex^28^: MED-EL Flex^28^ electrode array
Flex^Soft^: MED-EL Flex^Soft^ electrode array
RW: Round window
ERW: Extended round window
CS: Cochleostomy
