# Correction to: Electrode array design determines scalar position, dislocation rate and angle and postoperative speech perception

**DOI:** 10.1007/s00405-021-07215-4

**Published:** 2021-12-16

**Authors:** Manuel Christoph Ketterer, Antje Aschendorff, Susan Arndt, Rainer Beck

**Affiliations:** Department of Otorhinolaryngology-Head and Neck Surgery, Faculty of Medicine, Medical Center-University of Freiburg, University of Freiburg, Killianstrasse 5, 79106 Freiburg, Germany

## Correction to: European Archives of Oto-Rhino-Laryngology 10.1007/s00405-021-07160-2

In the original publication of the article, some values for the electrode arrays Flex^24^ and Flex^28^ (MED EL) have been interchanged in Tables 1 and 2 in the original manuscript. The authors apologize for that mistake. The correct Tables 1 and 2 are depicted below. The numbers used in the article itself were correct.

**Table 1:** Synopsis of study group (in total: *n* = 495)

Manufacturer (*n*)	Cochlear™: 327 MED-EL: 168
Electrode array (*n*)	Contour Advance (Cochlear™) (= CA): 143 CI 422/522/622 (Cochlear™) (= SSA): 162 CI 532/632 (Cochlear™) (= SMA): 22 Flex^24^ (MED-EL): 24 Flex^28^ (MED-EL): 129 Flex^Soft^ (MED-EL): 15
Side (*n*)	Left: 259 Right: 236
Age	52.7 years (min: 18.0; max: 86.2)

**Table 2:** Cochlear measurements (distance A and B), angular insertion depth, scalar position in total (SD = standard deviation) and distribution of the insertion technique

	Mean	SD	Minimum	Maximum
Distance A (mm)	9.92	0.86	7.4	12.2
Distance B (mm)	6.74	0.49	5.4	8.1
Insertion angle (°)	418.8	103.7	199	794
Scalar position in total (*n*)	ST: 434 (87.7%) TD: 32 (6.5%) SV: 25 (5%) VD: 4 (0.8%)			
Insertion technique in total (*n*) and percentage (%)	Electrode array	CS	RW	ERW
	CA (Cochlear™)	140/98%	3/2%	/
	SSA (Cochlear™)	47/29%	110/68%	5/3%
	SMA (Cochlear™)	12/54.5%	10/45.5%	/
	Flex^24^ (MED-EL)	4/16.6%	19/79.2%	1/4.2%
	Flex^28^ (MED-EL)	52/40.3%	72/55.8%	5/3.9%
	Flex^Soft^ (MED-EL)	12/80%	3/20%	/

*CS* cochleostomy, *RW* round window, *ERW* extended round window

